# Supplementary material for: Internal water channel formation in CXCR4 is crucial for Gi-protein coupling upon activation by CXCL12
Source: Commun Chem. 2020 Oct 8;3:133. doi: 10.1038/s42004-020-00383-0 (PMC9814148; doi:10.1038/s42004-020-00383-0)
Supplement: Supplementary file 1 — Supplementary Information [file 42004_2020_383_MOESM1_ESM.pdf]

Supporting information

Internal water channel formation in CXCR4 is crucial for G_i-protein coupling upon activation by CXCL12

Chun-Chun Chang[1,2,$], Je-Wen Liou[3,$], Kingsley Theras Primus Dass[4], Ya-Tzu Li[3], Shinn-Jong Jiang[3], Sheng-Feng Pan[3], Yu-Chen Yeh[3,4], and Hao-Jen Hsu[3,4*]

[1]*Department of Laboratory Medicine, Hualien Tzu Chi Hospital, Buddhist Tzu Chi Medical Foundation, Hualien 97004, Taiwan*
[2]*Department of Laboratory Medicine and Biotechnology, College of Medicine, Tzu Chi University, Hualien 97004, Taiwan*
[3]*Department of Biochemistry, School of Medicine, Tzu Chi University, Hualien 97004, Taiwan*
[4]*Department of Life Sciences, Tzu Chi University, Hualien 97004, Taiwan*

[$]C.-C. C. and J.-W. L. contributed equally to this work.

Supplementary Table S1. Top 10 poses of CXCL12-bound CXCR4 complex obtained from RDOCK calculation.							
**NO**	**ZRANK Score**	**E_vdw1 (kcal mol^{-1})**	**E_vdw2 (kcal mol^{-1})**	**E_elec1 (kcal mol^{-1})**	**E_elec12 (kcal mol^{-1})**	**E_sol (kcal mol^{-1})**	**E_RDOCK (kcal mol^{-1})**
1	-5.89	366.40	-126.49	-5.87	-44.66	0	-40.19
2	-16.78	11.93	-62.14	-5.20	-52.24	9.3	-37.71
3	11.78	104.21	-85.92	-4.32	-48.15	7.5	-35.84
4	-15.69	573.85	-85.43	1.50	-45.86	5.9	-35.37
5	-3.11	391.22	-49.60	-5.69	-55.69	17.8	-32.32
6	-66.31	-77.22	-86.54	-1.37	-42.07	7	-30.86
7	-11.60	-85.62	-94.49	-0.01	-47.73	14.7	-28.26
8	-57.41	-74.53	-86.83	-4.00	-39.51	9.4	-26.16
9	-23.38	-67.84	-62.67	-2.76	-39.72	10.6	-25.15
10	-23.42	-76.46	-94.22	-4.49	-35.46	7.3	-24.62

Pose 1 with the lowest RDOCK energy based on the ZDOCK module of Discovery Studio 3.5 (BIOVIA, http://accelrys.com) was selected for further MD simulation.

**Supplementary Table S2. Top 10 poses of CXCL12-bound mutant CXCR4 complex obtained from RDOCK calculation.**							
**NO**	**ZRANK Score**	**E_vdw1 (kcal mol^{-1})**	**E_vdw2 (kcal mol^{-1})**	**E_elec1 (kcal mol^{-1})**	**E_elec12 (kcal mol^{-1})**	**E_sol (kcal mol^{-1})**	**E_RDOCK (kcal mol^{-1})**
1	-49.05	-19.71	-107.03	4.56	-71.11	4.7	-59.30
2	-25.44	5.95	-148.66	-3.88	-62.30	-3.1	-59.17
3	-91.49	-84.56	-88.76	1.71	-61.96	5.4	-50.36
4	1.64	208.17	-142.74	-10.64	-49.96	-3.3	-48.26
5	-80.11	112.44	-78.35	-1.97	-53.39	0.1	-47.95
6	-8.23	117.74	-133.25	-1.18	-47.56	-4.5	-47.30
7	-10.68	160.33	-95.21	-9.13	-43.07	-8	-46.77
8	-31.86	107.52	-105.41	0.63	-54.67	3.2	-46.01
9	-5.02	905.78	-94.38	-9.13	-48.84	-1.6	-45.56
10	-39.47	52.08	-110.79	-3.51	-60.6	9.7	-44.89

Pose 1 with the lowest RDOCK energy based on the ZDOCK module of Discovery Studio 3.5 (BIOVIA, http://accelrys.com) was selected for further MD simulation.

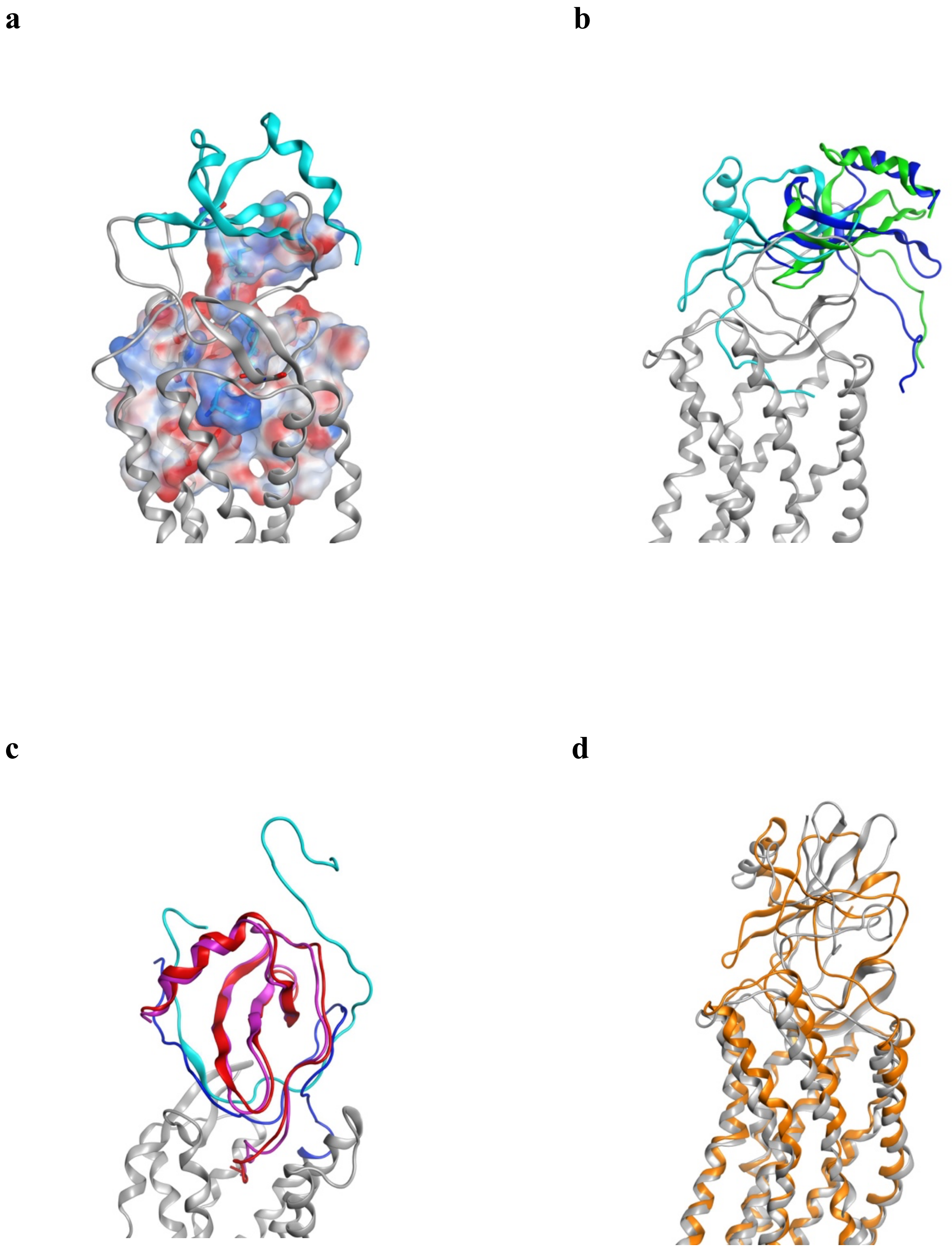
a
b
c
d

e

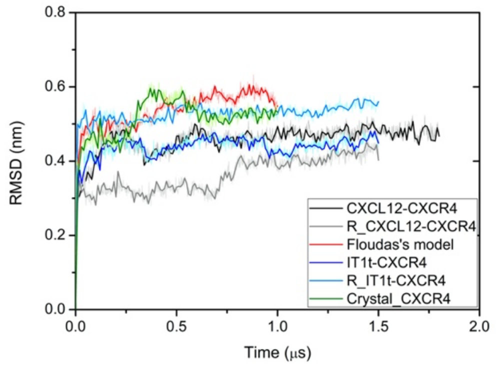

f

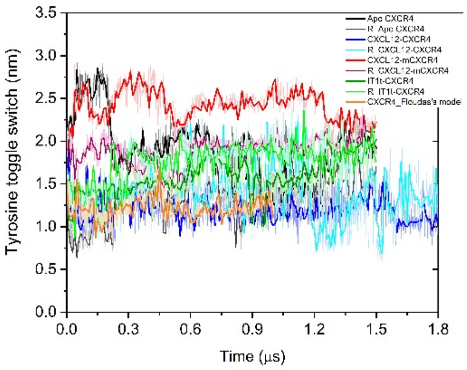

Supplementary Figure S1. Molecular docking results and superposition. (a) CXCL12 binding to mutant CXCR4. The surface charge distribution was calculated using the Poisson–Boltzmann equation. The complex is represented as a ribbon with agonist ligand CXCL12 colored cyan and mutant receptor mCXCR4 colored gray. Blue color corresponds to positive and red color to negative electrostatic potential. **(b)** Top 3 docking poses of CXCL12 docked to CXCR4 system. Cyan color CXCL12: pose1; blue color CXCL12: pose2; green color CXCL12: pose3. **(c)** Superposition of predicted binding interface for CXCL12–CXCR4 and solved NMR structure (PDB: 2N55). For NMR structures, the N-terminus of CXCR4 is represented as a cyan ribbon, and CXCL12 is represented as a purple ribbon. For the CXCL12–CXCR4 complex structure, CXCR4 is presented as a gray ribbon with N-terminus colored blue, and

CXCL12 is presented as a red ribbon. **(d)** The superposition of two CXCL12–CXCR4 complex structures: our model (orange color) and Volkman's proposed model (gray color), RMSD = 4.5 Å. **(e)** RMSD profiles with time. For CXCL12-bound and IT1t-bound CXCR4 systems, the replicate, crystal structure of IT1t–CXCR4 and another CXCL12−CXCR4 model proposed by Floudas *et al.*[16] were also performed MD simulations to indicate our simulations are comparable. **(f)** Tyrosine toggle switch profiles with time for all replicate simulation systems.

a

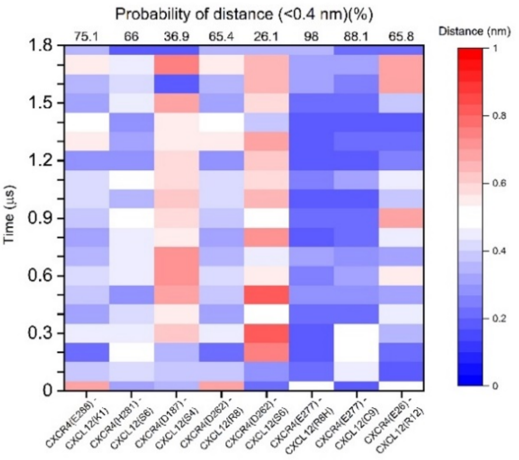

b

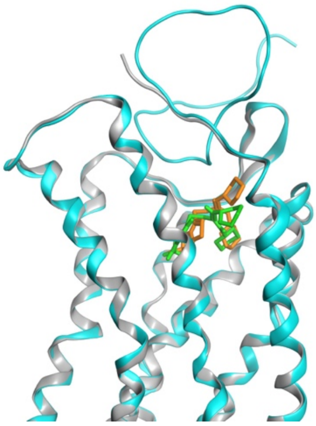

c

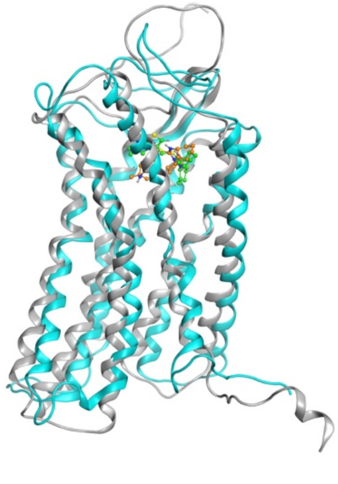

d

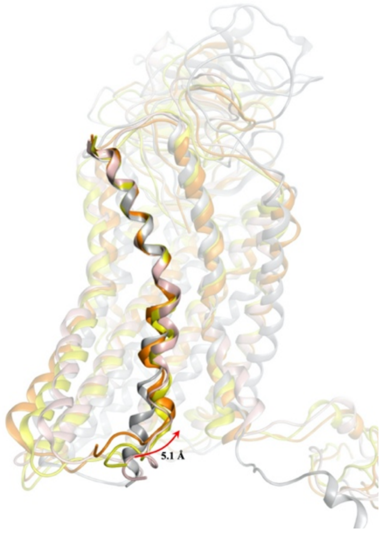

Supplementary Figure S2. Interaction heatmaps and superposition of various

CXCR4 complex systems at the different simulation time. (a) The interaction heatmaps with time for CXCL12 interaction with CXCR4. The probability of interaction distance less than 0.4 nm was also calculated. **(b)** The superposition of crystal IT1t–CXCR4 and redocked IT1t–CXCR4. Gray color: crystal CXCR4; cyan color: redocked CXCR4; orange color: crystal IT1t; green color: redocked IT1t. **(c)** IT1t-bound CXCR4 system. For the initial time, CXCR4 is represented as a gray ribbon, and IT1t is colored in orange. For the final simulation time, CXCR4 is represented as a cyan ribbon, and IT1t is colored in green. **(d)** CXCL12-bound mCXCR4 system. The superposition of CXCL12-bound mutant CXCR4 at different time frames showed the inward tilt 5.1 Å without affecting the ligand binding. Initial time: gray color; light pink color: 500 ns; yellow color: 1000 ns; orange color: 1500 ns.

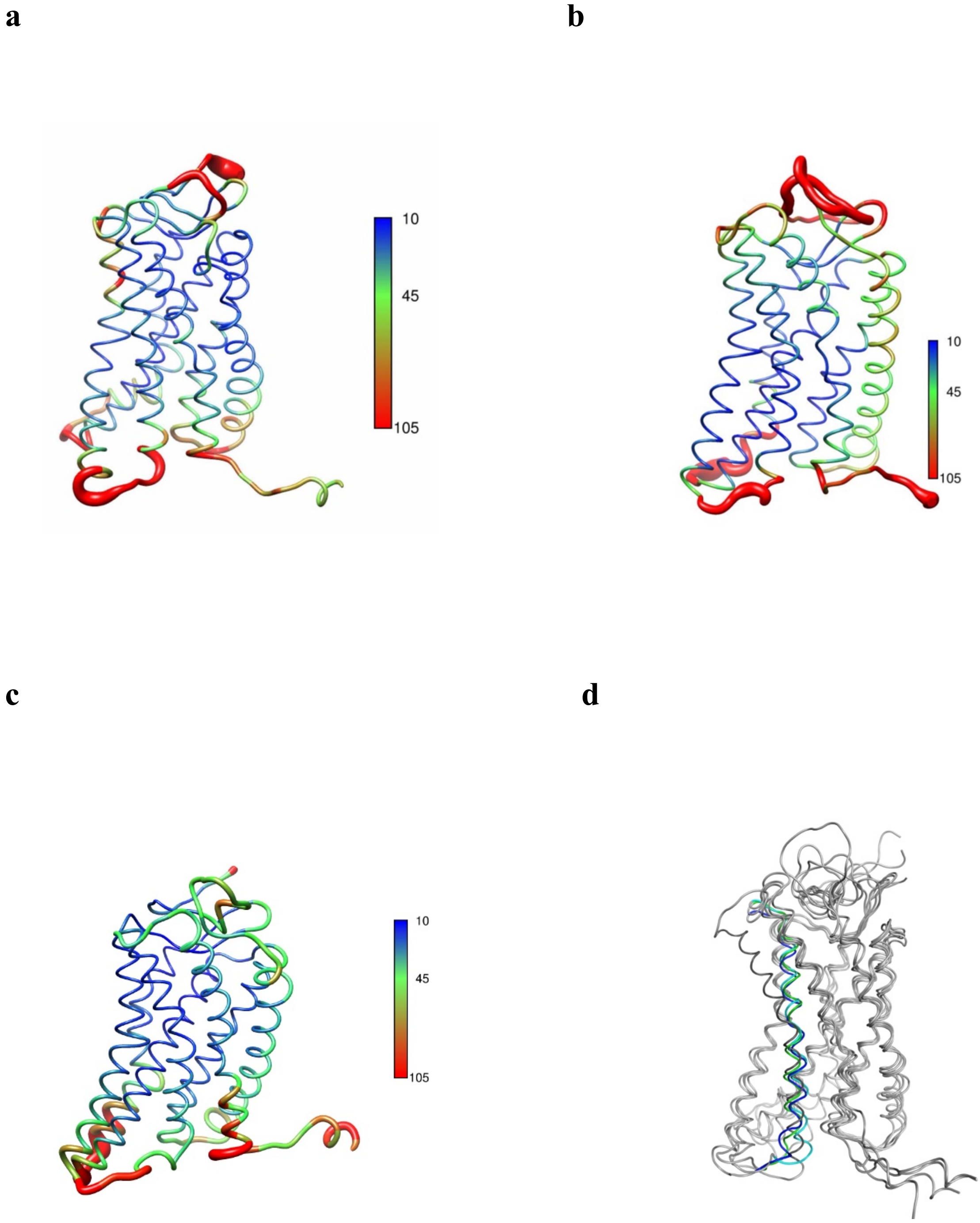
a
10
45
105
b
10
45
105
c
10
45
105
d

e

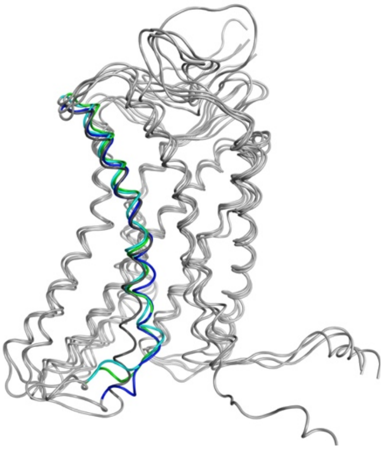

f

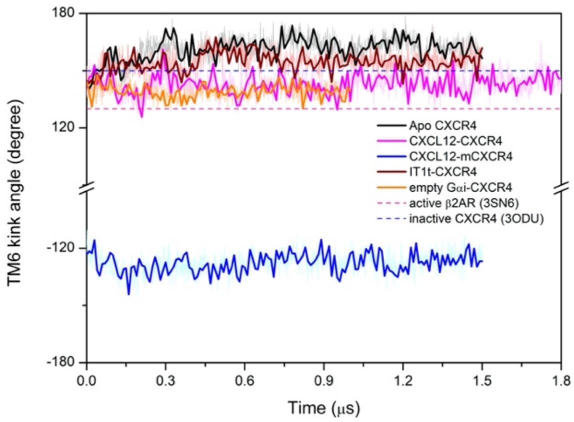

g

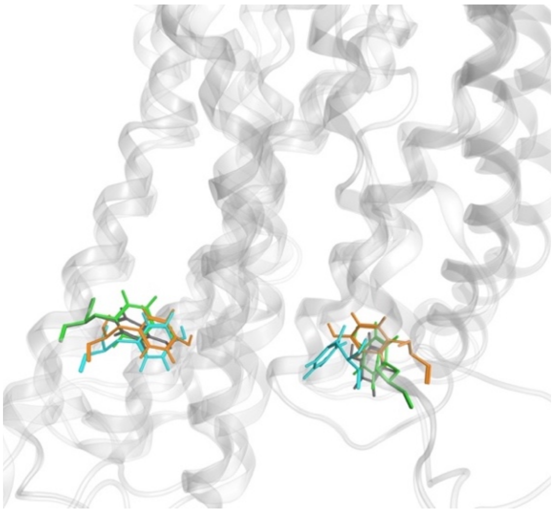

Supplementary Figure S3. B-factor and superposition of CXCR4 in various systems. (a)-(c) B-factor of C_α atoms of CXCR4 in various systems during the simulation time. In the color spectrum, red color represents more flexibility, and blue color indicates more rigidity. **(a)** Apo CXCR4. **(b)** Antagonist IT1t-bound CXCR4. **(c)**

CXCR4-bound mCXCR4. **(d)-(e)** Superposition of CXCR4 frames at various simulation time points. CXCR4 frames are represented as gray ribbons; dark gray is for TM6 at the initial time, blue is for TM6 at 500 ns, cyan is for TM6 at 1000 ns, and green is for TM6 at 1500 ns. **(d)** Apo CXCR4. **(e)** CXCR4-bound mCXCR4. **(f)** The kink of TM6 with time for various simulation systems. Black line is for apo CXCR4; purple line is for CXCL12–CXCR4; blue line is for mutant CXCR4 (mCXCR4); brown line is for antagonist IT1t–CXCR4; orange line is for CXCL12–CXCR4–empty (nucleotide free) $G_{\alpha i}$-protein; pink dash line is for active β_2AR–Gs complex structure (PDB: 3SN6); blue dash line is for inactive CXCR4 crystal structure (PDB: 3ODU). **(g)** Different CXCR4 structures at the final simulation time were superposed. Tyrosine residues were shown as sticks. For tyrosine, gray color: CXCL12−CXCR4 at the initial time; green color: IT1t–CXCR4 at the final time; cyan color: CXCL12−CXCR4 at the final time; orange color: CXCL12−CXCR4−$G_{\alpha i}$ at the final time.

a

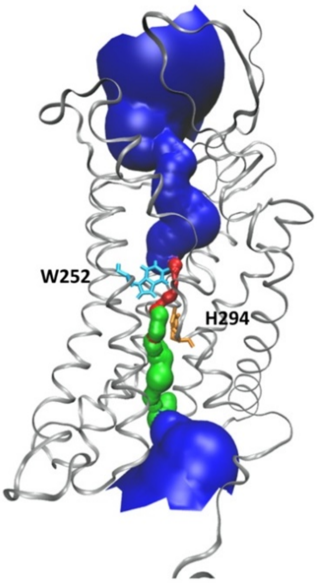

b

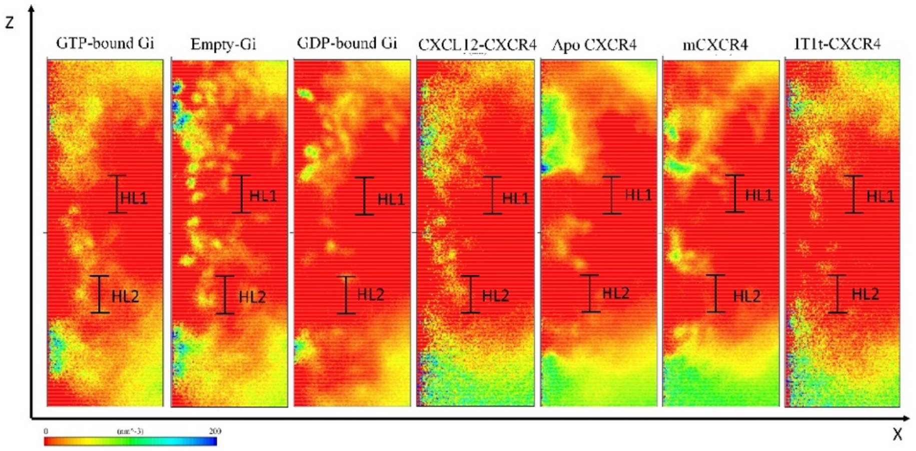

Supplementary Figure S4. Internal water pathway and water density maps of waters observed in the transmembrane region of respective systems. (a) The water

entrance pathway for CXCL12–CXCR4 system computed by HOLE program. Blue color is for pore radius >2.3 Å; green color is for pore radius 1.4~2.3 Å; red color is for pore radius <1.4 Å. Water molecules could easily enter from both EC and IC regions of CXCR4. **(b)** Water density distribution for various simulation systems were calculated, such as GTP-bound, empty, GDP-bound G_i states, CXCL12-bound CXCR4, apo CXCR4, CXCL12-bound mutant CXCR4, and IT1t-bound CXCR4 (from left to right). HL1, and HL2 are the hydrophobic layers in the transmembrane region. The empty G_i-bound CXCR4 was most activated in comparison to other systems, which formed a continuous water pathway that extended from the extracellular side to the intracellular side.

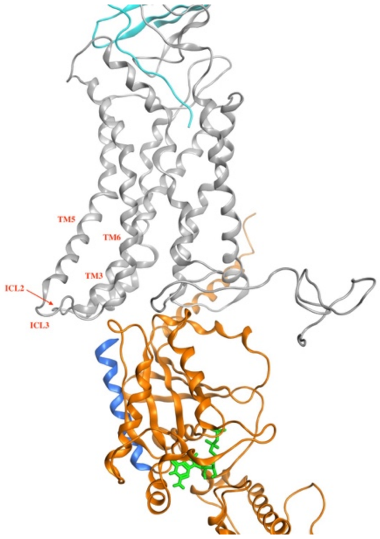

Supplementary Figure S5. Molecular docking of GDP-bound $G_{\alpha i}$-protein to CXCL12-bound CXCR4 receptor. The complex is represented as a ribbon, with CXCL12 colored cyan, CXCR4 colored gray, $G_{\alpha i}$-protein colored orange, and α5-helix colored blue, and GDP is represented by a green stick.

a

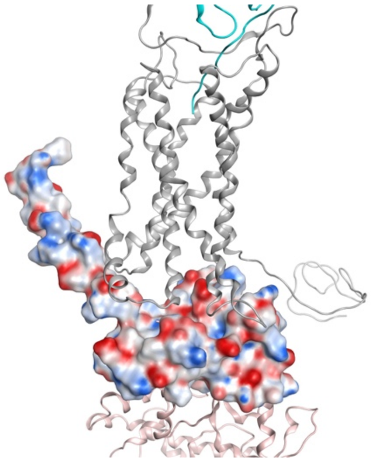

b

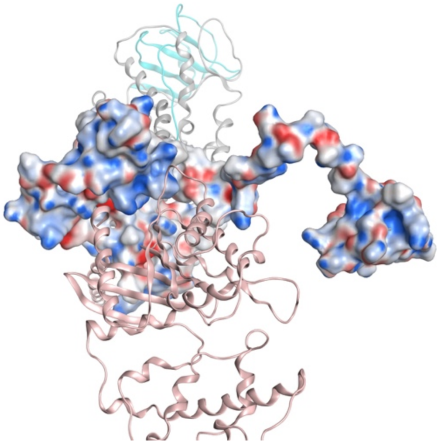

c

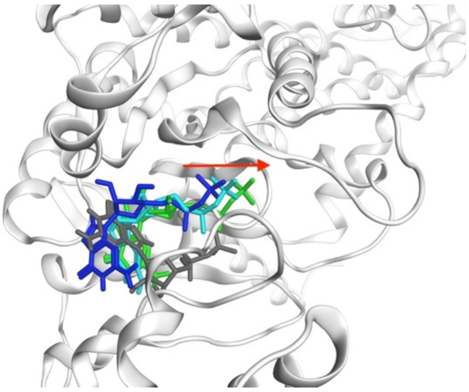

Supplementary Figure S6. Surface charge distribution and GDP movement for GDP-bound $G_{\alpha i}$-protein coupled with the pre-activated CXCR4 system. (a) and (b)

The complex is represented as a ribbon with agonist ligand CXCL12 colored cyan, receptor CXCR4 colored gray, and GDP-bound $G_{\alpha i}$-protein colored light pink. Blue color corresponds to positive and red color to negative electrostatic potential. **(c)** Superposition of GDP frames at various simulation time points. $G_{\alpha i}$-protein is represented by a gray ribbon, and GDPs are represented as sticks; dark gray color represents the initial time, blue color is at 500 ns, cyan color is at 1000 ns, and green color is at 1500 ns.

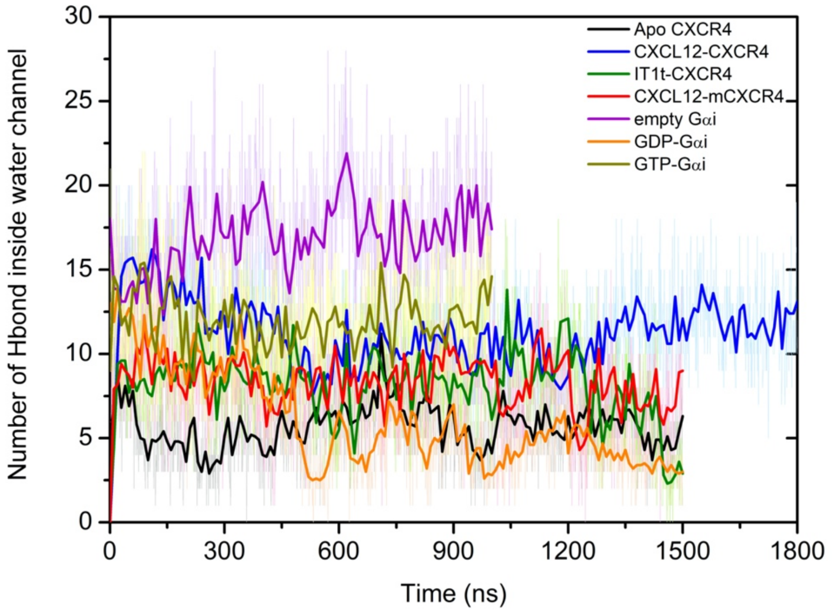

Supplementary Figure S7. Numbers of hydrogen bonding network with time for various simulation systems. Black line is for apo CXCR4; blue line is for CXCL12–CXCR4; green line is for antagonist IT1t–CXCR4; red line is for mutant CXCR4 (mCXCR4); purple line is for CXCL12–CXCR4–empty (nucleotide free) $G_{\alpha i}$-protein; orange line is for CXCL12–CXCR4–GDP-bound $G_{\alpha i}$-protein; dark green line is for CXCL12–CXCR4–GTP-bound $G_{\alpha i}$-protein.

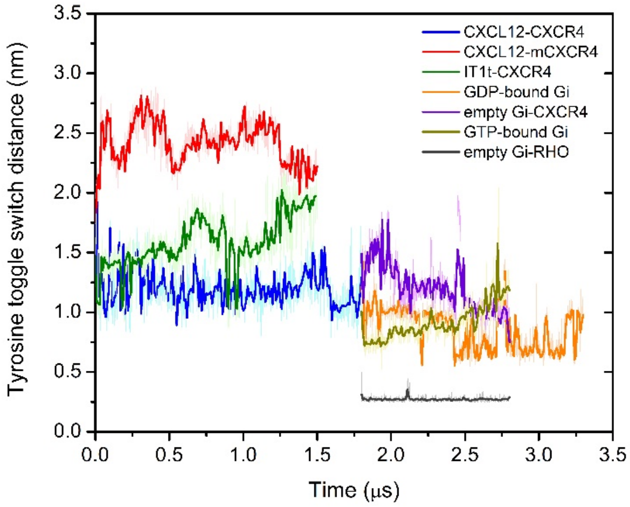

Supplementary Figure S8. Tyrosine toggle switch profiles with time for various G_i states-bound CXCR4 systems. Blue line is for CXCL12-bound CXCR4; red line is for CXCL12-bound mutant CXCR4; green line is for IT1t-bound CXCR4; purple line is for empty G_i-bound CXCR4; orange line is for GDP-bound G_i–CXCR4; olive line is for GTP-bound G_i–CXCR4; dark gray line is for empty G_i-bound RHO system. For various G_i states-bound CXCR4 systems, the simulation was defined to start from 1.8 μs. The switch distances (Y219$^{5.58}$-Y302$^{7.53}$) after G_i-protein binding were lower than that at inactive CXCR4 state.
