# Supplementary material for: Internal water channel formation in CXCR4 is crucial for Gi-protein coupling upon activation by CXCL12
Source: Commun Chem. 2020 Oct 8;3:133. doi: 10.1038/s42004-020-00383-0 (PMC9814148; doi:10.1038/s42004-020-00383-0)
Supplement: Supplementary file 2 — Reporting Summary [file 42004_2020_383_MOESM2_ESM.pdf]

nature research

Corresponding author(s): Hao-Jen Hsu

Last updated by author(s): Sep 11, 2020

Reporting Summary

Nature Research wishes to improve the reproducibility of the work that we publish. This form provides structure for consistency and transparency in reporting. For further information on Nature Research policies, see our Editorial Policies and the Editorial Policy Checklist.

Statistics

For all statistical analyses, confirm that the following items are present in the figure legend, table legend, main text, or Methods section.

n/a	Confirmed	
☐	☒	The exact sample size (n) for each experimental group/condition, given as a discrete number and unit of measurement
☐	☒	A statement on whether measurements were taken from distinct samples or whether the same sample was measured repeatedly
☒	☐	The statistical test(s) used AND whether they are one- or two-sided<br>*Only common tests should be described solely by name; describe more complex techniques in the Methods section.*
☒	☐	A description of all covariates tested
☒	☐	A description of any assumptions or corrections, such as tests of normality and adjustment for multiple comparisons
☐	☒	A full description of the statistical parameters including central tendency (e.g. means) or other basic estimates (e.g. regression coefficient) AND variation (e.g. standard deviation) or associated estimates of uncertainty (e.g. confidence intervals)
☒	☐	For null hypothesis testing, the test statistic (e.g. F, t, r) with confidence intervals, effect sizes, degrees of freedom and P value noted<br>*Give P values as exact values whenever suitable.*
☒	☐	For Bayesian analysis, information on the choice of priors and Markov chain Monte Carlo settings
☒	☐	For hierarchical and complex designs, identification of the appropriate level for tests and full reporting of outcomes
☒	☐	Estimates of effect sizes (e.g. Cohen's d, Pearson's r), indicating how they were calculated

Our web collection on statistics for biologists contains articles on many of the points above.

Software and code

Policy information about availability of computer code

Data collection	Molecular Operating Environment (MOE) software package MOE2016.08 (http://www.chemcomp.com). Discovery Studio 3.5 (BIOVIA, http://accelrys.com). GlycoBioChem PRODRG2 web server (http://davapc1.bioch.dundee.ac.uk/cgi-bin/prodrg). GROMACS 4.6.7 software package
Data analysis	GROMACS 4.6.7 software package; Origin 2020

For manuscripts utilizing custom algorithms or software that are central to the research but not yet described in published literature, software must be made available to editors and reviewers. We strongly encourage code deposition in a community repository (e.g. GitHub). See the Nature Research guidelines for submitting code & software for further information.

Data

Policy information about availability of data

All manuscripts must include a data availability statement. This statement should provide the following information, where applicable:

- Accession codes, unique identifiers, or web links for publicly available datasets
- A list of figures that have associated raw data
- A description of any restrictions on data availability

The data that support the findings of this study are available from the corresponding author upon reasonable request.

Field-specific reporting

Life sciences study design

All studies must disclose on these points even when the disclosure is negative.

Sample size	Atomic-level MD simulations were performed in various simulation systems. All the system sizes were listed in Table 2 of the manuscript. No real sample-size calculation was performed in our study.
Data exclusions	No data were excluded from the analyses.
Replication	For each simulation system, two replicates were successfully performed with the same condition.
Randomization	Randomization was not used in this study
Blinding	Blinding was not relevant to our study. Two replicate MD simulations were performed with different initial random numbers for each system. All MD trajectories were used for further analyses.

Reporting for specific materials, systems and methods

We require information from authors about some types of materials, experimental systems and methods used in many studies. Here, indicate whether each material, system or method listed is relevant to your study. If you are not sure if a list item applies to your research, read the appropriate section before selecting a response.

Materials & experimental systems

n/a	Involved in the study
☒	☐ Antibodies
☒	☐ Eukaryotic cell lines
☒	☐ Palaeontology and archaeology
☒	☐ Animals and other organisms
☒	☐ Human research participants
☒	☐ Clinical data
☒	☐ Dual use research of concern

Methods

n/a	Involved in the study
☒	☐ ChIP-seq
☒	☐ Flow cytometry
☒	☐ MRI-based neuroimaging
